# Supplementary material for: The interactome of intact mitochondria by cross-linking mass spectrometry provides evidence for coexisting respiratory supercomplexes
Source: Mol Cell Proteomics. 2017 Dec 8;17(2):216–32. doi: 10.1074/mcp.RA117.000470 (PMC5795388; doi:10.1074/mcp.RA117.000470)
Supplement: Supplemental Data [file supp_RA117.000470_133922_0_supp_23978_fzffwf.zip › spectra_annotation/mito_spectra_annotation/226-1-3-1-9-1.pdf]

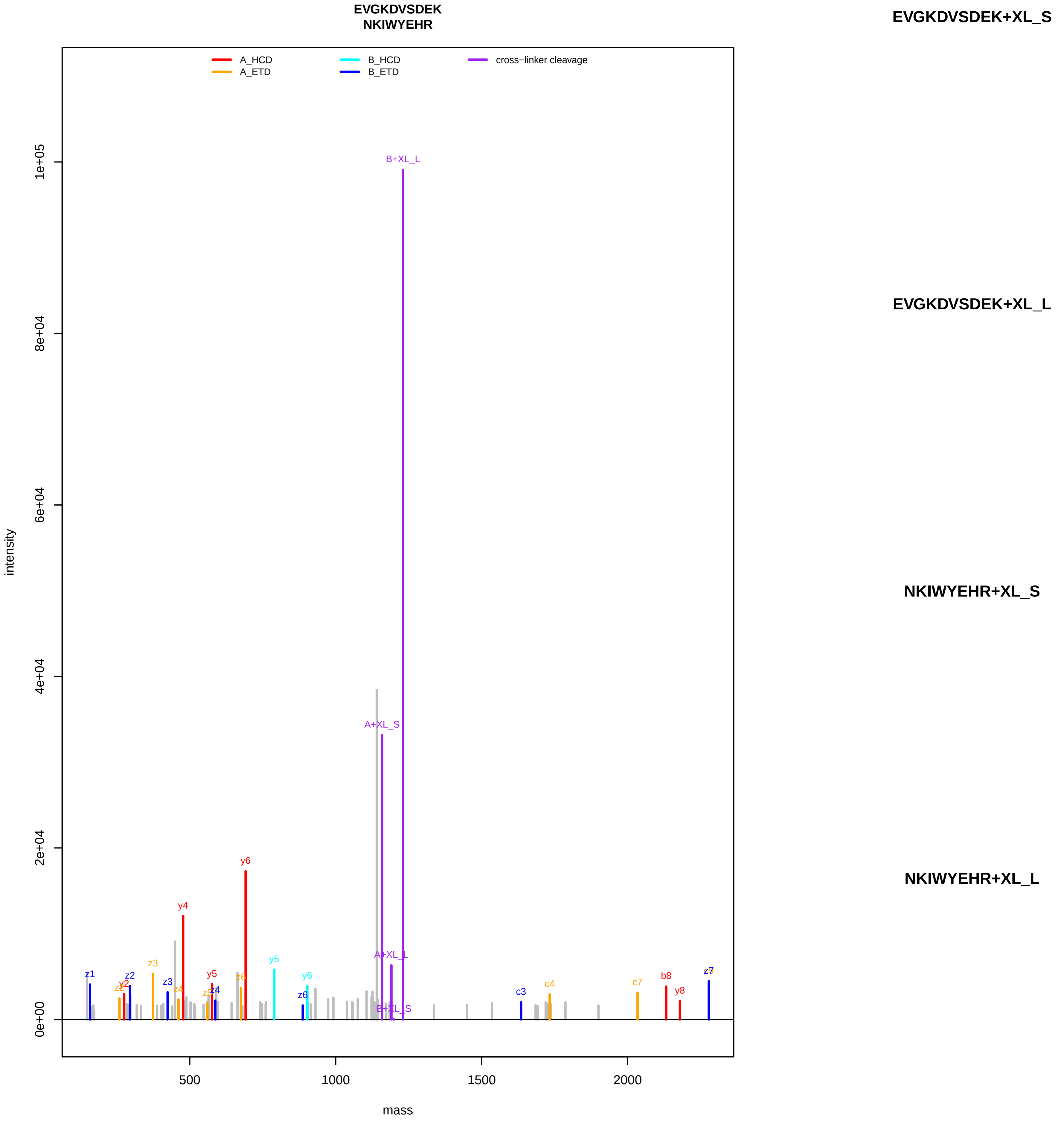
EVGKDVSDEK
NKIWYEHR
A_HCD
A_ETD
B_HCD
B_ETD
cross−linker cleavage
B+XL_L
A+XL_S
A+XL_L
B+XL_S
y2
y4
y5
y6
y8
b8
z1
z2
z3
z4
z5
z6
z7
c3
c4
c7
y5
y6
intensity
mass
0e+00
2e+04
4e+04
6e+04
8e+04
1e+05
500
1000
1500
2000
EVGKDVSDEK+XL_S
EVGKDVSDEK+XL_L
NKIWYEHR+XL_S
NKIWYEHR+XL_L
